# Redescription of *Alloceraea cretacea* (Acari: Ixodida) with an additional nymphal fossil added to this species: novel insights into the evolution of the Haematobothrion

**DOI:** 10.1017/S0031182026101826

**Published:** 2026-04

**Authors:** Lidia Chitimia-Dobler, Constantin Mey, Danilo Harms, Jörg U. Hammel, Jason Dunlop, Ulrich Kotthoff, Ben J. Mans

**Affiliations:** 1 Fraunhofer Institute for Translational Medicine and Pharmacology ITMP, Immunology, Infection and Pandemic Research IIP, Munich, Germany; 2 Experimental Parasitology, Department of Veterinary Sciences, Faculty of Veterinary Medicine, Ludwig-Maximilians-Universität, LMU, Munich, Germany; 3 Universität Hamburg, Institute for Geology, Hamburg, Germany; 4 Museum of Nature Hamburg, Leibniz-Institute for the Analysis of Biodiversity Change (LIB), Hamburg, Germany; 5 Helmholtz-Zentrum Hereon, Institute of Materials Physics, Geesthacht, Germany; 6 Museum für Naturkunde, Leibniz Institute for Evolution and Biodiversity Science, Berlin, Germany; 7 Epidemiology, Parasites and Vectors, Agricultural Research Council-Onderstepoort Veterinary Research, Onderstepoort, South Africa; 8 Department of Life and Consumer Sciences, University of South Africa, Florida, South Africa; 9 Department of Zoology and Entomology, University of the Free State, Bloemfontein, South Africa

**Keywords:** *Alloceraea cretacea*, Burmese amber, fossil tick nymphs, *Haemaphysalis* (*Alloceraea*) *cretacea*, Haematobothrion, paleontology

## Abstract

A fossil assigned to the extant ixodid genus *Haemaphysalis* was previously described from the Late Cretaceous (ca. 99 Ma) Burmese amber. *Haemaphysalis* (*Alloceraea*) *cretacea* was considered the oldest, and only, fossil representative of this genus. Significant criticism was raised regarding possible misidentification of this amber fossil. Microtomography of the original holotype and new material allows new perspectives on this controversy. A new fossil nymph is described and both fossils are considered *Alloceraea cretacea* comb. nov. nymphs based on a series of morphological characters: no genital aperture, eyeless, 11 festoons, coxa I simple with a short, wide triangular spur. Specific morphological features for the ‘structurally primitive’ *Alloceraea* are discussed and include palpi elongate, with long setae on palps, the hypostome dorsally longer than the chelicera and the corona visible distally and with a specific distribution of the denticles. *Alloceraea* and its sister genus *Archaeocroton* (that includes amber fossil taxa), share a common ancestor with *Haemaphysalis* sensu stricto, indicating a minimum divergence time of at least 100 MYA for these lineages. In addition, a fossil of *Bothriocroton* has also been described from Burmese amber. This genus with *Cryptocroton* groups basal to the *Alloceraea*/*Archaecroton-Haemaphysalis* assemblage forming a monophyletic clade named Haematobothrion. A synthesis of the fossil information and the current systematic understanding of the Haematobothrion allows new hypotheses about the origins of this group and the various lineages it comprises. Notably that the major Haematobothrion lineages originated and diverged during the time period when the Burma terrane was migrating from Australia to Asia.

## Graphical abstract

**Figure:**
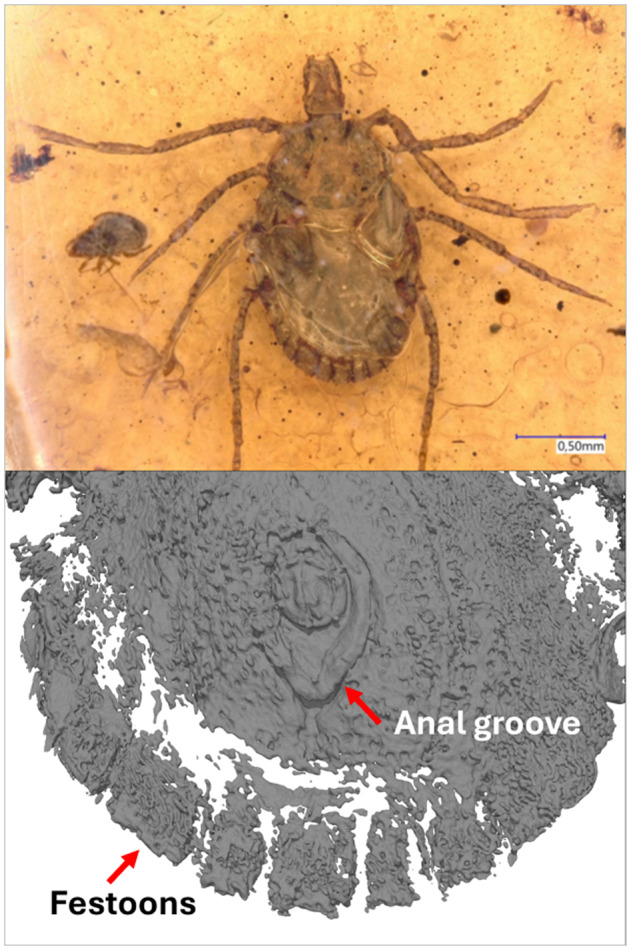

## Introduction

In the hard tick family Ixodidae, there are currently 763 recognized species that includes 18 extant genera (Guglielmone et al., 2023; Barker et al., 2024; Kelava et al., 2024; Apanaskevich et al., 2025). For 5 of these genera, extinct species have been recognized in Burmese amber, namely *Alloceraea* (this study), *Amblyomma, Archaeocroton, Bothriocroton* and *Ixodes* (Chitimia-Dobler et al., 2017, 2022, 2023). Two extinct hard tick genera have also been described from Burmese amber (Upper Cretaceous, ca. 99 mya), namely *Compluriscutula* Poinar and Buckley, 2008 and *Cornupalpatum* Poinar and Brown, 2003 (Poinar and Brown, 2003; Poinar and Buckley, 2008). A *Cornupalpatum burmanicum* Poinar and Brown, 2003 nymph entangled in a pennaceous feather suggested that it may have fed on feathered avian or non-avian dinosaurs (Peñalver et al., 2017). This finding is supported by the description of an additional *C. burmanicum* female associated with a dinosaur feather in Burmese amber (Chitimia-Dobler et al., 2022). Numerous larvae of *Compluriscutula vetulum* Poinar and Buckley, 2008 were found in the Burmese amber, which suggests that it may have been relatively common in the Burmese amber forest, perhaps preferring arboricolous hosts (Chitimia-Dobler et al., 2024a). In addition to these recent species there is a single fossil from the mid-Cretaceous Burmese (or Kachin) amber of Myanmar which was initially described as *Haemaphysalis (Alloceraea) cretacea* Chitimia-Dobler, Pfeffer and Dunlop, 2018 (Chitimia-Dobler et al., 2018).

*Haemaphysalis* CL Koch, 1844 is the second largest genus in the Ixodidae, comprising 173 described species (Guglielmone et al., 2023; Chitimia-Dobler et al., 2024b; Robbins et al., 2025). This genus occurs in all 6 zoogeographic regions, but the greatest species diversity is found in south-eastern Asia while species numbers are much lower in the Nearctic and the Neotropics (Guglielmone et al., 2023). The best represented area for *Haemaphysalis* is the Oriental region, with 64 species.

*Haemaphysalis* was traditionally divided into 11 subgenera that can be broadly classified into 3 groups: (1) the structurally primitive, (2) structurally intermediate, and (3) structurally advanced species (Hoogstraal and Kim, 1985). ‘Structurally primitive’ *Haemaphysalis* comprised of 4 subgenera: *Alloceraea* Schulze, 1919, *Allophysalis* Hoogstraal, 1959, *Aboimisalis* Santos Dias, 1963, and *Sharifiella* Santos Dias, 1958 (Hoogstraal and Kim, 1985; Geevarghese and Mishra, 2011). Using mitochondrial systematics, *Allophysalis* and *Aboimisalis* was shown to group within *Haemaphysalis* sensu stricto (Kelava et al., 2024), leaving only *Alloceraea* and *Sharifiella* as potential basal lineages. Similarly, *Sharifiella* was recently elevated to genus level since mitochondrial systematics indicated grouping outside *Haemaphysalis*, within the Rhipicephalinae (Apanaskevich et al., 2025). As such, it does not share a relationship with any of the Haemaphysalinae. Conversely, *Alloceraea* was recently shown to group within its own clade within the Haematobothrion and was re-validated as a distinct genus based on mitochondrial systematics (Kelava et al., 2024). Molecular analysis defined the Haematobothrion as being comprised of 5 genera, *Bothriocroton* and *Cryptocroton* forming one potentially basal clade, while *Archaecroton* and *Alloceraea* forms a sister clade to *Haemaphysalis* sensu strictu (Barker et al., 2024; Kelava et al., 2024).

*Alloceraea* contains species with distributions mainly in Asia and the Orient and only 1 species is found in Europe. In detail, *Alloceraea aponommoides* (Warburton, 1913) comes from India, *Alloceraea inermis* (Birula, 1895) from Europe and Asia, *Alloceraea kitaokai* (Hoogstraal, 1969) and *Alloceraea primitiva* (Teng, 1982) from Asia, and *Alloceraea colasbelcouri* (Santos Dias, 1958) and *Alloceraea kolonini* (Du, Sun, Xu and Shao, 2018) are from the Orient (Feider, 1965; Filippova, 1997; Geevarghese and Mishra, 2011; Guglielmone et al., 2014).

The elevation of *Alloceraea* to genus level implies that *H. cretaceae* should also be transferred to *Alloceraea* as *Alloceraea cretacea* comb. nov. However, some studies have questioned the assignment of this fossil to *Haemaphysalis* and *Alloceraea* (Guglielmone et al., 2020; Kelava et al., 2024). Here, we re-describe *A. cretacea* to address the critiques raised previously regarding its taxonomic placement and describe a new representative fossil nymph of the same species using µCT data and digital imaging. In addition, implications of the fossil record for the evolution of the Haematobothrion lineage, composed of *Alloceraea, Archaeocroton, Bothriocroton*, and *Haemaphysalis* are discussed.

## Materials and methods

Two fossil ticks, both nymphs, originate from the collection of Patrick Müller bearing the original inventory numbers BUB 990 and BUB 2779. Both specimens are from Burmese amber that come from the Hukawng Valley in northern Myanmar and have been deposited in the Museum für Naturkunde, Berlin. Light microscopy images were taken with a Keyence VHX-900 Microscope (Keyence Itasca, IL, USA) with 100× to 200× magnification) for the morphological description. In addition, high-resolution μCT scans were carried out with synchrotron radiation at the German Electron Synchrotron (DESY). A precise comparison was carried out using the models subsequently created by editing the 3D digital models. Synchrotron radiated micro-computed tomography (SRmCT) was performed at the imaging beamline P05 (IBL) operated by Helmholtz-Zentrum Hereon at PETRA III at Deutsches Elektronen-Synchrotron in Hamburg, Germany (Greving et al., 2014; Wilde et al., 2016). Scanning occurred using a custom-built CMOS camera (Lytaev et al., 2014) with a photon energy of 18 keV. Raw projections were binned twice, and a reconstruction was made by applying a transport of intensity phase retrieval approach using the Filtered Back Project algorithm, implemented in a custom reconstruction pipeline (Moosmann et al., 2014) with Matlab (Math-Works) and Astra Toolbox (Palenstijn et al., 2011; van Aarle et al., 2015, 2016). Segmentation was performed in Amira (v.6.0.1; FEI Company, Hillsboro, OR, USA) by selecting the specimen in every 20th or 30th image to generate a label, which was then used to interpolate all intervening images with the program Biomedisa (Lösel et al., 2023: https://biomedisa.org/). A 3D-rendering was then exported and deposited in MorphoSource (https://www.morphosource.org/; Project ID: 000583128).

Moreover, 5 extant *Haemaphysalis* from different life stages (larva, nymph, 2 female and male) and species were scanned and used for comparative purposes to define morphological characteristics for this genus. This include *Haemaphysalis* (*Allophysalis*) *danieli* Černý and Hoogstraal, 1977 female (Pakistan), *Haemaphysalis* (*Haemaphysalis*) *concinna* Koch, 1844 female (Collections of the Museum of Nature Hamburg, Germany), *Haemaphysalis* (*Rhipistoma*) *leachi* (Audouin, 1826) male (Collections of the Museum of Nature Hamburg, Germany), *Haemaphysalis* (*Ornithophysalis*) *eleonorae* Chitimia-Dobler, Mans and Saratsis, 2024 nymph (Greece), and *A. inermis* larva (Italy).

Recent comparative material of ticks is lodged at the following institutions: Leibniz Institute for Evolution and Biodiversity Science (MfN), Museum of Nature Hamburg – Zoology (ZMH), Leibniz Institute for the Analysis of Biodiversity Change, Private collection of Lidia Chitima-Dobler (Munich, Germany).

## Results

### Systematic palaeontology

Ixodida, Leach, (1815)

*Alloceraea*, Schulze, (1919)

*Alloceraea cretacea* comb. nov. Chitimia-Dobler, Pfeffer and Dunlop, 2018

*Material*: Holotype BUB990 and additional material BUB2779 (coll. P. Müller). Burmese amber, Myanmar, Late Cretaceous (Cenomanian).

*Emended diagnosis*: Body oval-elongate, scutum broader than long with margins broadly rounded, palpi elongate and clavate, hypostome with well-marked transversal ridges of denticles connected by crenulations and 4 stout denticles on each side, cornua and eyes absent, 11 festoons, anal groove ‘V’ shape, spiracle plates oval-elongate, coxae with a broadly sub-triangular spur, trochanter spurs lacking.

*Remarks*: Herein, the holotype was re-analysed using novel synchrotron tomographic scanning images taken at the Deutsches Eletronen-Synchrotron (DESY). Moreover, another specimen (additional material BUB2779) from the same original collection was investigated. As the new specimen shares the same morphology as the holotype, we will further refer only to the new morphological features and clarify the controversial discussions and critique raised against the original type description.

The hypostome is spatulate and longer than chelicera and hypostomal denticles can be seen dorsally and clarified with the DESY images and videos. The hypostomal denticles have a special arrangement: 8 well-marked transversal ridges of 6 (proximal) to 8–10 (distal) denticles connected by crenulations and on each side 4 stout denticles; not separated in the middle (1A, B). Such structures are hard to see using light microscopy or reproduced in drawings, especially of nymphs. Such crenulations were also observed in DESY scans of the extant species *H.* (*A.*) *danieli, H.* (*H.*) *concinna, H.* (*R.*) *leachi, H.* (*O.*) *eleonorae* and *A. inermis* (2A, B, C, D, E). The crenulations are less developed in larval instars and become more pronounced during subsequent life stages. The amber fossils have no spur on the palps, but they do have an anterior process and a small blunt spur angular on the basis capituli (3A, B). DESY scans of the fossils show that the scapulae are in fact blunt and round and not long and sharp as it was originally described (Chitimia-Dobler et al., 2018). The sharp aspect described in the original description derived from a spur on the distal part of coxae I in both specimens (BUB-990, BUB-2779) (Figure 4). Each coxa has a broadly sub-triangular spur extending slightly (I–III) beyond the coxal margin, IV spur small (Figure 5). Anal groove has a ‘V’ shape, although not well visible (Figure 6).

**Figure 1.**
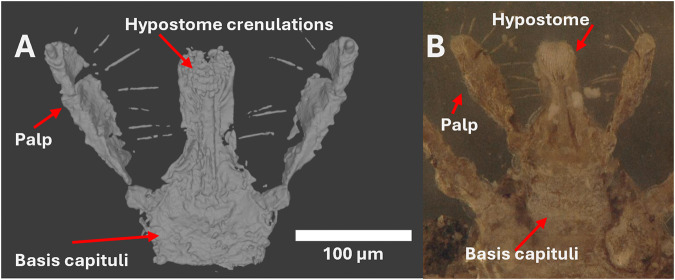
*Alloceraea cretacea* (BUB-990) – A, capitulum ventral (DESY image), hypostome crenulations, palp, and basis capituli are marked with red arrows; B, capitulum ventral (light microscopy Keyence VH900), hypostome crenulations, palp and basis capituli are marked with red arrows. Figure 1 long description.

**Figure 2.**
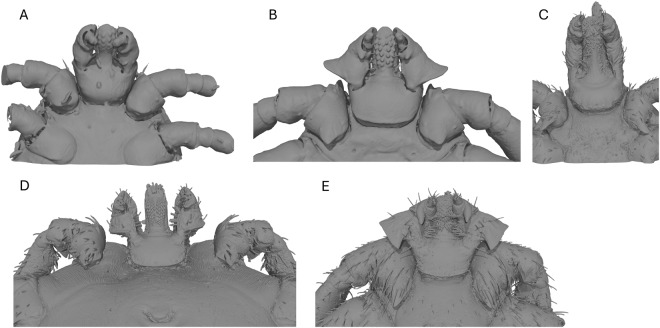
Ventral view of capituli and coxa I of different life stage and species of extant *Haemaphysalis*, focusing on hypostome structure: A, *Alloceraea inermis* larva (Italy); B, *Haemaphysalis* (*Ornithophysalis*) *eleonorae* nymph (Greece); C, *Haemaphysalis* (*A.*) *Danieli* female (Pakistan); D, *Haemaphysalis* (*Haemaphysalis*) *concinna* female (Collections of the Museum of Nature Hamburg, Germany) and E, *Haemaphysalis* (*Rhipistoma*) *leachi* male (Collections of the Museum of Nature Hamburg, Germany). Figure 2 long description.

**Figure 3.**
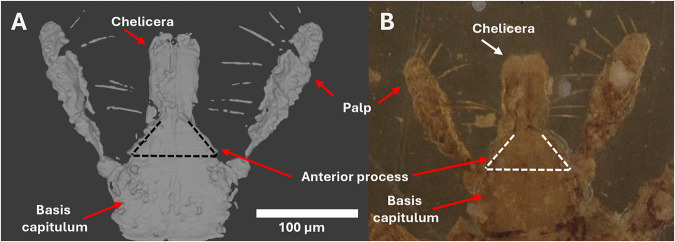
*Alloceraea cretacea* (BUB-990) – A, capitulum dorsal (DESY image), chelicera, palp, anterior process and basis capituli are marked with red arrows; B, capitulum dorsal (light microscopy Keyence VH900), chelicera, palp, anterior process and basis capituli are marked with red arrows. Figure 3 long description.

**Figure 4.**
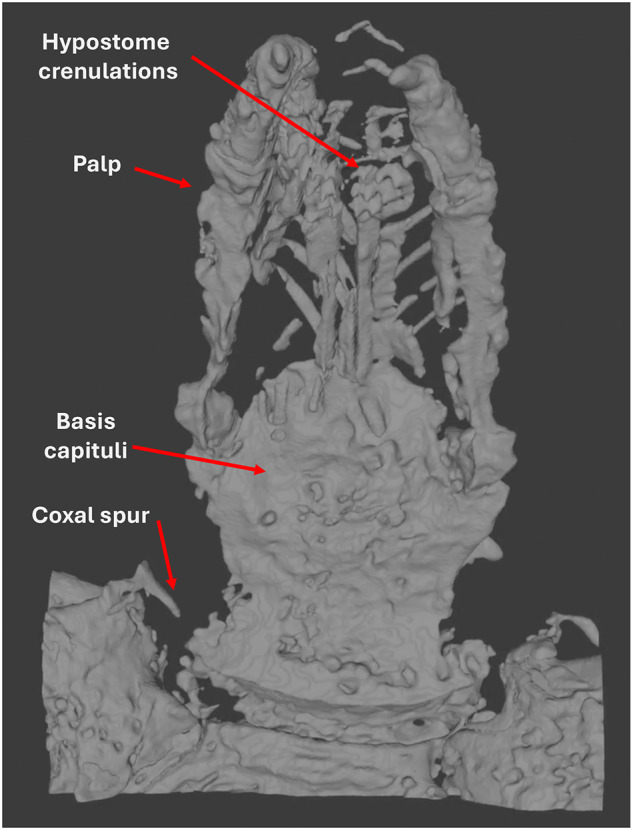
*Alloceraea cretacea* (BUB-2779) – capitulum ventral (DESY image), hypostome crenulations, palp, basis capituli and spur on the coxa I are marked with red arrows. Figure 4 long description.

**Figure 5.**
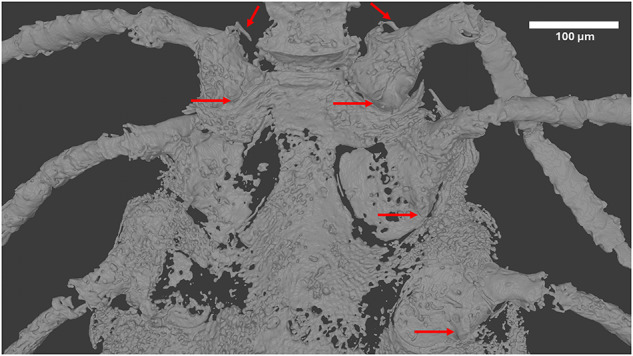
*Alloceraea cretacea* (BUB-2779) – coxae (DESY image), internal spur of coxae I-III and apical spurs are marked with red arrows. Figure 5 long description.

**Figure 6.**
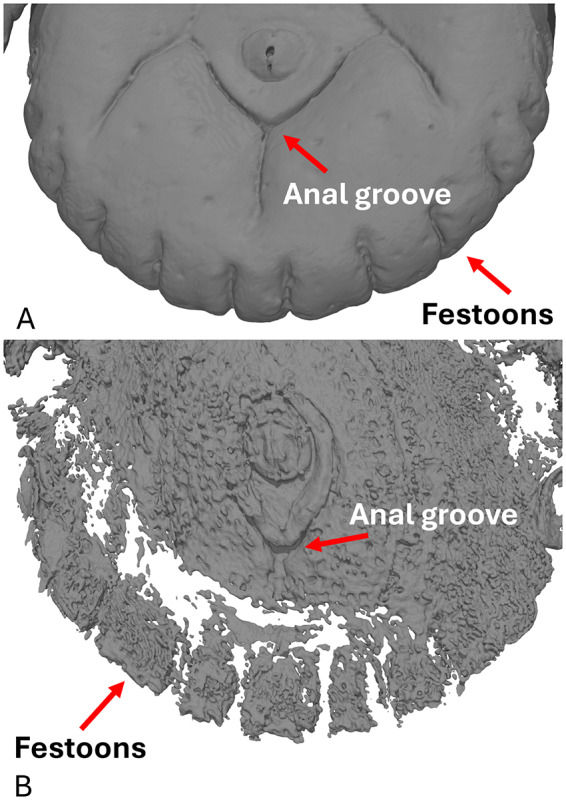
Comparison of the anal grooves – A, extant *Haemaphysalis* (*Ornithophysalis*) *eleonorae* nymph; B, *Alloceraea cretacea* (BUB-2779) nymph. Festoons are also clearly seen for both nymphs. Figure 6 long description.

**Figure 7.**
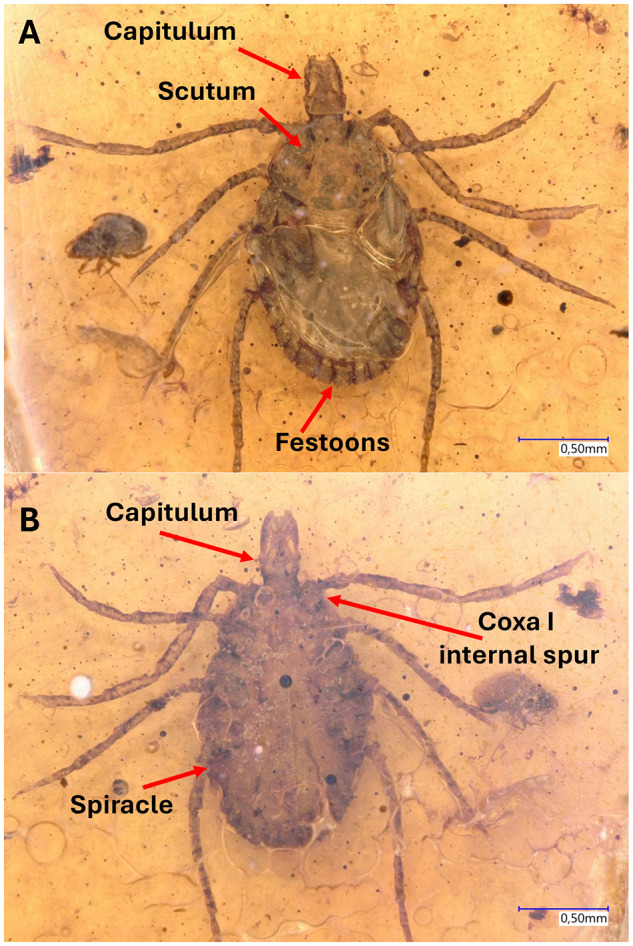
*Alloceraea cretacea* (BUB-2779) – A, dorsal view, capitulum, scutum, festoon, marked with red arrows; B, ventral view, capitulum, coxa I internal spur and spiracle, marked with red arrows (light microscopy Keyence VH900). Figure 7 long description.

## Discussion

The current study adds a new nymphal fossil which we assign to *A. cretacea* (7A, B). The elongate body, typical hypostomal structure, very long setae on the palps, a well-developed dorsal anterior process of the palps that extends laterally from the basis capituli, simple coxa I, long spur on the distal part of coxae I, a small sub-triangular spur, and the absence of laterally projections of the basis capituli are the most relevant features to identify the 2 fossils as conspecific and to argue that they can be assigned to *Haemaphysalis* sensu lato based on general characteristics typical for this genus such as the lack of eyes, the number of festoons, and the ‘V’ anal groove shape which place them within the Metastriata and not Prostriata.

## Alloceraea *classification and dubious taxonomic assignments*

We note in this context that the *A. cretacea* fossil described by Chitimia-Dobler et al. (2018) elicited significant critique in the literature. Guglielmone et al. (2020) commented that the morphological structures from Chitimia-Dobler et al. (2018) do not support inclusion in the genus *Haemaphysalis*. They argue that while the authors indicate that the basis capituli is slightly wider than long or 2.6 times wider than long, the accompanying figures show a basis capituli that is obviously longer than broad. Also, that the second article of the palps is 1.6 times longer than the third article, but that the figures show a second article almost 4 times longer than the third article. They then cite Hoogstraal and Kim (1985) to indicate that ‘the most basic criterion of the 17 “primitive” *Haemaphysalis* is the presence, in each stage, or only in larvae and nymphs, of a lateral convexity of the basis capituli or of a projection from each side of the basis capituli’, indicating that this is not met in the nymph described by Chitimia-Dobler et al. (2018). The current study argues that Guglielmone et al. (2020) did not recognize that the measurements were made from the palp insertion and did not include the basis capituli anterior process. The anterior process was not included in the anterior description, as it never appeared in the *Haemaphysalis* species description or in other species. Furthermore, the basis capituli posterior have straight margins, while dorsally the lateral margins display concavity and a blunt slightly angular shape, which does not extend over the scapula and is only slightly visible ventral (1A, 3A). The lateral margin concavity was observed in larva and females of *A. inermis*, which is considered a distinct morphologic feature. The second concavity is on the side of the anterior process, at the level of the first palpal segment, a character rendering the fossil different from *A. inermis*. Hoogstraal (1966a) stipulated that the capitulum of larva and nymphs is more like that of *Rhipicephalus* and *Dermacentor* immatures than of most other *Haemaphysalis*. The *Alloceraea* also do not have lateral projections, either dorsally or ventrally, on the basis capituli. For example, see *A. aponommoides* (Geevarghese and Mishra, 2011). In the current study, this type of projection was not observed on basis capituli or palps of *A. inermis* scanned larva (2A). Another general morphological aspect for the ‘structurally primitive’ group was the absence of cornua. This morphological aspect is characteristic only for *Alloceraea* and not found in *Haemaphysalis* sensu stricto (Hoogstraal, 1966a; Geevarghese and Mishra, 2011).

## Alloceraea – *A ‘structurally primitive’ genus*

Recently, *Alloceraea* was considered the only remaining ‘structurally primitive’ genus, given that molecular data place the other ‘primitive’ groups in *Haemaphysalis* sensu stricto (Kelava et al., 2024). This study also critiqued Chitimia-Dobler et al. (2018) raising a number of morphological issues, notably that the palps from *A. cretacea* are considerably narrower and longer, with exceptionally long setae compared with other known nymphs of *Alloceraea*. That the first palpal segment of *A. cretacea* is more developed than any other known *Alloceraea* species. That the basis capitulum is different in shape from other *Alloceraea* with a distinct anterior process, absent in extant *Alloceraea*. That the shape of the scutum and the long and sharp scapulae are different from extant *Alloceraea*. That the legs are long and slender, while extant *Alloceraea* have short and robust legs. That the idiosoma of the unfed fossil is narrowly oval whereas extant *Alloceraea* this is widely oval. That the hypostome, the shape of the dorsal basis capitulum, the coxal spurs on coxa, anal groove and eyes is not visible.

To address these quite substantial critiques, we re-evaluated the amber specimens using tomographic images and videos of both available specimens. None of the morphological aspects discussed in Chitimia-Dobler et al. (2018) are included here, since repetition is not necessary. Our focus here is on new morphological aspects of the 2 specimens as they relate to the critiques raised and towards the classification of *A. cretacea* within the ‘structurally primitive’ genus *Alloceraea*.

*Haemaphysalis* ticks are generally small, inornate, and have short mouthparts: the brevirostrata condition. Hoogstraal and Kim (1985) divided them into the ‘structurally primitive’, ‘structurally intermediate’ and ‘structurally advanced’ groups. It was emphasized that ‘the most basic criterion of the 17 “primitive” *Haemaphysalis* is the presence, in each stage, or only in larvae and nymphs, of a lateral convexity of the basis capitulum, or of a projection from each side of the basis capitula’. Kelava et al. (2024) indicated that the basis capituli in *A. cretacea* has a very distinct anterior process, whereas all extant ex-*Alloceraea* species lack this. Here, we would add that some *Haemaphysalis* species, especially from the earlier ‘structurally primitive’ group, do have an anterior process, small in *A. aponommoides* and more evident in other *Haemaphysalis* (*Allophysalis*) species, e.g. *H*. (*Allophysalis*) *garhwalensis* (Geevarghese and Mishra, 2011), *H*. (*Allophysalis*) *pospelovashtromae* Hoogstraal, 1966 (Hoogstraal, 1966a).

We accept that the exceptionally long setae in the 2 amber fossils are not comparable with any extant *Alloceraea* nymphs, but beyond *A. inermis*, other species with long setae include *H. (A.) pospelovashtromae* (Hoogstraal, 1966a). Long setae may therefore be a morphological aspect for some *Haemaphysalis* species. Many of the earlier ‘structurally primitive group’ species have nymphal palps described as elongate and clavate (Hoogstraal and Kim, 1985), similar to the amber fossils. Another aspect should be noted here, the position of the nymphal palps in extant ‘structurally primitive’ species which extend posterior from the basis capituli and laterally from the basis capituli in the fossil (Figure 3).

The precise dental formula of the hypostome in the new specimen was not entirely clear under light microscopy. However, numerous denticles and a corona were distal visible. The hypostome is spatulate and longer than the chelicera, and hypostomal denticles can be seen dorsally (Figures 1 and 3) as clarified by the DESY images and videos. Additionally, in *A. cretacea* (e.g. BUB-990) the hypostomal denticles have a special arrangement: 8 well-marked transversal ridges of 6 (proximal) to 8—10 (distal) denticles connected by crenulations and on each side 4 stout denticles; not separated in the middle (1A). This morphology is difficult to observe under light microscopy or to reproduce in drawings, especially for nymphs. However, in Geevarghese et al. (2009) the drawing of *Haemaphysalis* (*Kaiseriana*) *aculeata* Lavarra, 1904 partially shows the denticles connected by crenulations in the middle, even if the dental formula is 2/2. To clarify this, we chose 5 extant species: *H.* (*A.*) *danieli, H.* (*H.*) *concinna* and *H.* (*R.*) *leachi, H.* (*O.*) *eleonorae* and *A. inermis* and scanned them at the DESY (2A, B, C). We conclude now that crenulations, or denticles connected by crenulations, are a specific morphological feature for *A. cretacea*, which could be an ancestral state for *Haemaphysalis* sensu lato, as crenulations were observed in scanned extant species. *Alloceraea inermis* larvae has less crenulations maybe due to its 2/2 denticles, the host and the short time feeding (Nosek, 1973; Filippova, 1997). *Haemaphysalis* (*O.*) *eleonorae* nymphs show increased crenulations compared to larvae and explains why its denticle formula looks confusing (Chitimia-Dobler et al., 2024c). Female *H.* (*A.*) *danieli* presents more evident crenulations, and female *H.* (*H.*) *concinna* shows some reminiscence of crenulations in the middle and distal of the hypostome, while male *H.* (*R.*) *leachi* has evident crenulations. It seems that the presence of crenulations on the hypostome is an ancestral morphological aspect maintained during the evolution and development of the genus *Haemaphysalis* sensu lato which we consider it to be associated with the hosts of the different life stages and species.

‘Structurally primitive’ *Haemaphysalis*, and the other non-rhipicephaline ixodids, differ distinctly from other haemaphysaline species. It has been suggested that life stages from *Alloceraea* have a laterally convex or otherwise laterally projecting basis capituli, lacking cornua, and elongate (clavate) and compact palpi, but not basolaterally salient, lacking a ventral spur (Geevarghese and Mishra, 2011). Our fossils have *Alloceraea* characteristics, such as elongate palpi, absence of a ventral spur on the palps and lack cornua, but can be differentiated from the living species. *Allophysalis* immatures have short, broadly angular basis capituli, but the palpi remain elongate as in *Alloceraea*. Bearing a small ventral spur on the palpi suggests *Allophysalis* is close to the structurally advanced *Ornithophysalis* subgenus (Geevarghese and Mishra, 2011). Presence of spurs on the palps is considered to be a bird-parasitizing or primitive mammal parasitizing adaptation for the structurally advanced subgenus *Ornithophysalis* (Hoogstraal and Kim, 1985; Geevarghese and Mishra, 2011).

Larvae of the subgenus *Herpetobia*, which is classed as ‘structurally intermediate’, lack cornua and a discrete coxal spur which is taken as evidence of reptile-parasitizing progenitors (Hoogstraal and Kim, 1985; Geevarghese and Mishra, 2011). Immatures and adults from the *Haemaphysalis* subgenus have palps which are only slightly advanced, compact, slightly elongate, either broader posteriorly (as in *Herpetobia* nymphs), or elongate with moderate flange, characterizing *Herpetobia* and the ‘structurally primitive’ lineages (Hoogstraal and Kim, 1985).

Regarding the long and sharp scapulae of *A. cretacea*, the synchrotron scans show that the original description was incorrect with the scapulae blunt and round. The ‘sharp’ aspect was misinterpreted from a spur on the distal part of coxae I. Interestingly, such a spur, albeit smaller, was observed on *A. inermis* larva, which could be a characteristic for *Alloceraea*.

Regarding coxal spurs, scans show that each coxa bears a broadly sub-triangular spur extending slightly (I–III) beyond the coxal margin, with the IV spur small. The *A. inermis* larva has the same spurs on their coxae, just smaller. The anus and anal groove were unclear in the original description due to an artefact or insufficient contrast. In the new fossil nymph (BUB2779), the anal groove can be seen as a ‘V’ shape with a little tail (Figure 6). The anal groove matches the condition observed in *Haemaphysalis* sensu lato, including *Alloceraea*. Some authors describe the anal groove as ‘V’ shaped (Feider, 1965), while other authors do not comment on the shape but their figures indicate a ‘Y’ shaped anal groove (Hoogstraal, 1966b; Hoogstraal et al., 1971). The ‘Y’ shaped anal groove can be observed in the *H.* (*O.*) *eleonorae* nymph we used for comparison (Figure 6).

The typical hypostomal structure, simple coxa I, long spur on the distal part of coxae I with a small triangular spur are the most relevant features to define *Haemaphysalis* sensu lato together with being eyeless, presence of festoons, and a ‘V’ shaped anal groove. In conclusion, we remain confident that both fossils are best placed in the genus *Alloceraea.*

## *Taxonomic re-definition of* Alloceraea

Based on the new findings presented above, we redefine the morphological features which characterize *Alloceraea*, primarily based on a combination of (1) oval body shape, (2) the absence of eyes, (3) the presence of festoons, (4) a dental formula with 8–10 denticles in a file, (5) absence of lateral projections of the basis capituli, (6) a small dorsal anterior process of basis capituli, (7) lack of cornua, (8) the absence of trochanter spurs and (9) segment III of palps lacking a ventral spur and segment IV being an apical pit. Regarding character 5: in contrast to *Alloceraea, Allophysalis* and *Aboimisalis* have lateral protections of the basis capituli on larvae and nymphs and cornua in adults.

### Evolution and biogeography of Haematobothrion lineages

Developments in our knowledge of the Burmese amber fossils since the initial description of *A. cretacea* allow new insights regarding the evolution of the Haematobothrion lineages. The presence of extant *Bothriocroton* in Australasia (Klompen et al., 2002; Guglielmone et al., 2023) as well as fossils in Burmese amber (Chitimia-Dobler et al., 2023), suggest that the Burma terrane (BT) was colonized by this lineage before it rifted from Proto-Australia; events that may have occurred between ∼170 and 150 MYA (Westerweel et al., 2025). Its absence in Asia is consistent with the hypothesis that the lineage went extinct during the BT journey that lasted from ∼150 to 40 MYA (Westerweel et al., 2025). Conversely, the presence of living *Alloceraea* in the Palearctic and Oriental regions and as fossils in Burmese amber, but the absence of this genus in Australasia (Guglielmone et al., 2023), could imply that the lineage ancestral to *Alloceraea, Archaeocroton* and *Haemaphysalis* evolved after rifting from Australia. In this scenario, further divergence of lineages that formed *Haemaphysalis* and *Alloceraea-Archaeocroton* occurred during the migration of the BT towards Asia, followed subsequent divergence of *Alloceraea* and *Archaeocroton*. It is plausible that the ancestral *Achaeocroton* lineage dispersed from the BT terrane to the Polynesian islands to eventually end up as a single extant species in New Zealand (Heath, 2006), also suggesting significant extinction events in the Cenozoic. *Cryptocroton* shows a deep divergence with *Bothriocroton* (Barker et al., 2024). This likely indicates that divergence occurred on the Australasian mainland, which may suggest that *Cryptocroton* fossils may also be found in Burmese amber. This divergence of the lineages is consistent with our current understanding of Haematobothrion systematics (Barker et al., 2024; Kelava et al., 2024).

Molecular dating using mitochondrial genome data also supports this scenario with previous divergence times for *Bothriocroton* and the rest of the Haematobothrion estimated at ∼155 MYA (Mans et al., 2019; Chitimia-Dobler et al., 2023), the split between *Haemaphysalis* and *Alloceraea-Archaeocroton* at ∼151 MYA and *Alloceraea* and *Archaeocroton* at ∼139 MYA (Mans et al., 2019; Chitimia-Dobler et al., 2023). The timing for these events thus correlates with divergence after the BT rifting and during the BT journey. It would also imply that *Alloceraea* and *Haemaphysalis* survived the journey from Australia to Asia to colonize the Palearctic Region (including Europe, most parts of Asia and northern Africa). Evidence that other lineages survived the BT journey has been offered for spiders and fresh-water mussels (Bolotov et al., 2022; Wood and Wunderlich, 2023). This would make ticks another lineage to survive the passage with the implication that vertebrate hosts also had to survive to maintain these lineages.

In the case of *Alloceraea* the colonization event was not very successful, with only 6 extant species described to date, of which 5 are restricted to the Orient and 1 to the Palearctic (Guglielmone et al., 2023). *Haemaphysalis*, however, is considered one of the more successful genera, comprising ∼21% of ixodid tick species of which ∼47% occur in the Oriental region (Guglielmone et al., 2023). This would suggest divergence of the lineage once it reached the new continent and potentially found new hosts. This scenario is congruent with other suggestions for an origin for *Haemaphysalis* in the Oriental region with subsequent dispersal to other biomes (Geevarghese and Mishra, 2011). It is also supported by the basal position of the Oriental lineages, with more derived lineages from the Australasian region in a more terminal position (Kelava et al., 2024). This hypothesis explains the phylogenetic pattern seen for the Haematobothrion lineage and finally makes sense of some puzzling geographic patterns when systematic sister-group relationships are considered. If *A. cretacea* does not belong to *Alloceraea* sensu stricto, as some authors suggested, but rather represents a direct ancestor to *Haemaphysalis*, or groups within the general Haematobothrion clade, this would still not change the general hypothesis that links the geographically distinct *Bothriocroton, Cryptocroton, Alloceraea, Archaeocroton* and *Haemaphysalis* together via vicariance and divergence precipitated by the BT rifting and journey to Asia.

### Summary

The present study showcases the usefulness of Burmese amber fossils to help understand deep evolutionary relationships between modern tick genera. In the same context, Burmese amber fossils have provided insights into the evolution of prostriates (Chitimia-Dobler et al., 2023) and the Nuttalliellidae family (Chitimia-Dobler et al., 2024c). The implication of the current study is that Burmese fossils will continue to contribute to our understanding of tick evolution, in this case specifically that fossils belonging to *Haemaphysalis* sensu stricto may be expected to be found in Cretaceous ambers as well.

## Figure 1 Long description

The image A showing a grayscale micrograph on a black background. A central upright structure is labeled “Hypostome,” with an arrow pointing to the upper end. The top edge of the central structure is labeled “Hypostome crenulations,” with an arrow pointing to the upper margin. Two lateral structures extend outward from the central structure; the left lateral structure is labeled “Palp,” with an arrow pointing to it. The lower central region is labeled “Basis capituli,” with an arrow pointing to the base. A scale bar at the lower right reads “100 µm.” The image B showing a brown-toned micrograph on a dark background. A central upright structure is labeled “Hypostome,” with an arrow pointing to the upper portion. A lateral structure on the left is labeled “Palp,” with an arrow pointing to it. The lower central region is labeled “Basis capituli,” with an arrow pointing to the base.

Navigate back to Figure 1.

## Figure 2 Long description

A five-part scientific figure arranged in two rows, with labels A, B, C on the top row and D, E on the bottom row. Each part shows a ventral view centered on the capituli and the first pair of coxae, with the hypostome in the middle. A shows a compact central capituli with two short lateral appendages and two coxa I structures extending outward. B shows a broader capituli with wider lateral extensions than A and coxa I extending to both sides. C shows a narrower, more elongated central capituli region than A and B, with coxa I partly shown at the sides. D shows a larger field of view than A to C, with the capituli near the top center and coxa I extending outward; the lateral appendages appear thicker and more textured. E shows a wide capituli with prominent lateral extensions and many short, hair-like projections around the margins; coxa I extends outward on both sides. No scale bar or measurement text is shown.

Navigate back to Figure 2.

## Figure 3 Long description

Image A shows a dorsal view of the capitulum with chelicera, palp, anterior process and basis capituli marked with arrows. The scale bar indicates 100 micrometers. Image B also shows a dorsal view of the capitulum with chelicera, palp, anterior process and basis capituli marked with arrows. Both images highlight similar anatomical features with different imaging techniques.

Navigate back to Figure 3.

## Figure 4 Long description

The 3D scan displays a detailed view of a structure with several anatomical features marked by arrows. The hypostome crenulations are indicated at the top, showing intricate patterns. Below, the palp is marked, highlighting its position in the structure. The basis capituli is identified in the central region, providing a reference point for the anatomy. At the bottom, the coxal spur is marked, showing its distinct shape and location. The scan provides a comprehensive view of these features, emphasizing their spatial relationships and structural details.

Navigate back to Figure 4.

## Figure 5 Long description

Insufficient visual information to describe this element accurately.

Navigate back to Figure 5.

## Figure 6 Long description

The image A showing a grayscale micrograph with a curved outer edge and a central circular structure near the top. Two red arrows with white text labels are present: one arrow labeled “Anal groove” points toward a curved groove near the center; one arrow labeled “Festoons” points toward the scalloped outer margin. The image B showing a grayscale micrograph with a broad curved band of textured material and a lighter curved region inside it. Two red arrows with white text labels are present: one arrow labeled “Anal groove” points toward a curved groove near the inner side of the band; one arrow labeled “Festoons” points toward the scalloped outer margin.

Navigate back to Figure 6.

## Figure 7 Long description

The image A showing an arthropod body with multiple legs on a pale brown background. Black text labels read Capitulum, Scutum and Festoons, each with a red arrow pointing to the corresponding body region. A blue scale bar at the lower right reads 1.50mm. The image B showing an arthropod body with multiple legs on a pale brown background. Black text labels read Capitulum, Coxal internal spur and Spiracle, each with a red arrow pointing to the corresponding body region. A blue scale bar at the lower right reads 1.50mm.

Navigate back to Figure 7.
